# When scientific constraints and real-world implementation collide: Lessons from a hybrid type 3 study of a digital, direct-to-consumer HIV prevention program

**DOI:** 10.21203/rs.3.rs-9202620/v1

**Published:** 2026-04-23

**Authors:** Kathryn R. Macapagal, Krystal L. Madkins, Ashley Knapp, Manuel Hurtado, Mariajosé Paton, Bryant Norton, Josephine Owusu, Isaac Greenawalt, Dennis H. Li, Brian Mustanski

**Affiliations:** Northwestern University; Northwestern University; Northwestern University; Northwestern University; Northwestern University; Northwestern University; University of Illinois at Chicago; Long Island University; Northwestern University; Northwestern University

**Keywords:** Digital health interventions, Direct-to-consumer (DTC) interventions, hybrid effectiveness-implementation study, HIV prevention

## Abstract

**Background:**

Digital HIV prevention interventions (DHIs) are efficacious in increasing prevention behaviors, and delivering them direct-to-consumer (DTC) expands their reach. However, few have successfully moved rigorously-studied DHIs from research to public health practice. Hybrid type 3 effectiveness-implementation studies promise to approximate more naturalistic settings and accelerate translation from research to practice, and few DTC DHIs have been tested this way. This study describes lessons learned from a hybrid type 3 study of a DTC DHI for young men who have sex with men (YMSM) ages 18–29 called Keep It Up! (KIU!) 3.0.

**Methods:**

KIU! 3.0 was initially designed as a cluster-randomized hybrid type 3 implementation-effectiveness study across 44 United States counties with high HIV incidence among YMSM (22 DTC; 22 implemented in community-based organizations). The DTC strategy relied on online recruitment, at-home HIV/STI testing, and centralized intervention delivery. Over the course of the trial (October 2019-March 2023), we adapted our implementation four times in response to recruitment and retention challenges. Data sources included enrollment logs, advertising expenditures, participant communications, and internal documentation, which are used to characterize recruitment, costs, and recruitment/retention patterns.

**Results:**

Due to challenges in recruitment and retention, four major changes were made over the course of the trial: 1) streamlining enrollment procedures, including shortening screening and verification steps, 2) modifying eligibility criteria, including expanding age limits, removing sexual risk requirements, and including gender-diverse participants, 3) introducing and increasing financial incentives for intervention completing and follow-up measures, and 4) shifting from county-level to nationwide recruitment. Following this fourth change in March 2021, enrollment increased by 301%, and cost per enrolled participant decreased. Ultimately 1,468 participants were enrolled nationwide. Nevertheless, retention and return of at-home STI test kits remained challenging, and guaranteed incentives increased the prevalence of imposter participants.

**Conclusion:**

Implementing a DTC DHI within a hybrid type 3 study required balancing pragmatic implementation goals with effectiveness outcomes measurement. Future hybrid studies of DTC digital health interventions should consider eligibility criteria, incentive structures, outcomes measurement strategies, and the distinction between research and service components to better align research with real-world implementation contexts.

## Introduction

In the United States (U.S.), men who have sex with men (MSM) accounted for 67% of new HIV diagnoses in 2022 (CDC, 2025a), most of which were within young MSM aged 13–34 (CDC, 2025a). Digital HIV interventions (DHIs; e.g., interactive websites, smartphone apps, text messaging programs) tailored to their needs are efficacious in reducing HIV transmission risk behavior and sexually transmitted infection (STI) incidence and increasing health promotive behaviors (e.g., pre-exposure prophylaxis [PrEP] use, HIV testing). DHIs can reduce barriers to care if delivered “direct-to-consumer” (DTC) rather than through intermediaries, such as healthcare providers or community health centers (Li et al., 2019; Nguyen et al., 2019; Zhang et al., 2021). Currently, 29 DHIs are listed in a compendium of “best practices” in HIV risk reduction (CDC, 2025b), but none are being implemented in public health practice. This is because despite significant federal investment in research to *develop and test* DHIs, relatively little research has attempted to *implement* them in real-world settings, and those who have reported significant obstacles (Hermes et al., 2019; Li et al., 2024).

Researchers have studied DHI implementation in two main ways: via healthcare or community-based organizations (CBOs) trained to offer the intervention to clients and via a DTC model, where an organization hosts and/or markets the intervention to people who can self-enroll (Benbow et al., 2025; Mustanski, Benbow, et al., 2025b; Mustanski, Macapagal, et al., 2025; Mustanski, Saber, Macapagal, et al., 2023). Most literature on DHI implementation has focused on healthcare settings with “captive audiences” of existing patients or clients. In contrast, a DTC model requires the implementer (e.g., digital health company, health department) to focus on creating significant demand for the intervention (via marketing, incentives, etc.) among people who may not perceive the need for one (Aref-Adib et al., 2019; Germain et al., 2021; Matson et al., 2025; Ross et al., 2018). Yet this may be challenging for preventive interventions as individuals are often less motivated to address health problems before they arise (Hardcastle et al., 2015; Hermes et al., 2019). In traditional effectiveness trials, demand creation activities are ancillary and often not detailed in publications, but in implementation studies they are essential in supporting DTC intervention delivery. How well these strategies operate in a “real-world” setting has not been well-documented.

## The Keep It Up! (KIU!) intervention

We developed KIU!, a self-guided DHI for YMSM that is among the CDC’s best-evidence HIV prevention programs (Centers for Disease Control, 2024) and based on the Information-Motivation-Behavioral Skills theory of health behavior change (Fisher et al., 1994). KIU! was originally designed to be delivered in CBOs after YMSM tested HIV-negative to encourage them to “keep up” their preventive behaviors (KIU! 1.0; (Mustanski et al., 2013). A randomized controlled trial (KIU! 2.0) examined the effectiveness of CBO-based delivery in three U.S. cities and piloted a nationwide DTC strategy where staff recruited YMSM through online advertising, shipped HIV/STI test kits to participants, and granted access to KIU! when a participant tested HIV-negative (Mustanski et al., 2017). KIU! reduced self-reported condomless anal sex and gonorrhea and chlamydia incidence by 40% versus an HIV information-only control arm (Mustanski et al., 2018).

From 2019 to 2023, we conducted a hybrid type 3 implementation–effectiveness study (KIU! 3.0) comparing the CBO and DTC strategies; the protocol and findings are described elsewhere (Mustanski, Benbow, et al., 2025b; Mustanski, Saber, Jones, et al., 2023). We chose this design to approximate real-world implementation; however, the constraints of a research study necessarily limits flexibility. As few studies explore DTC delivery of DHIs, this trial offered a unique opportunity to examine KIU!’s effectiveness in a more naturalistic setting, but also examine what worked (and did not) regarding implementation.

This paper describes the initial implementation plans for the KIU! 3.0 DTC strategy and how they evolved because of recruitment challenges and COVID-19 pandemic disruptions. We also discuss tensions between the research elements necessary to measure KIU!’s effectiveness and pragmatic elements more aligned with real-world delivery. Our goal is to offer a “behind the scenes” look at the study operations, decision making, and lessons learned in demand creation, and to share ideas about what can/cannot change during a hybrid 3 study of a DHI which can inform future pragmatic studies and reduce implementation barriers (Li et al., 2019).

## Methods

### Initial study design and context

The KIU! 3.0 intervention consisted of five “episodes” (three reflecting the main intervention content and two boosters), each containing a webseries, didactics, and activities to augment learning and engagement that totaled ~ 1.5h. After episodes 1–3, participants waited 8h until the next episode. A feature of earlier KIU! versions, this break was based on guidelines to enhance information retention in multimedia learning (Clark & Mayer, 2008; Mayer, 2017, 2016). Booster content in episodes 4 and 5 (at 3- and 6-months post-intervention, respectively) reinforced learning. Participants completed a final survey and STI testing at 12 months post-intervention.

The study initially proposed a cluster-randomized design to compare the implementation and effectiveness of the KIU! CBO versus DTC strategies in 44 U.S. counties with high HIV incidence among YMSM (Mustanski, Saber, Jones, et al., 2023). Enrollment goals were 100 per county (N = 4400), and county-level randomization was selected to mirror how HIV public health funding often works and to compare implementation in high-incidence jurisdictions outlined in the federal Ending the HIV Epidemic initiative (*EHE Priority Jurisdictions*, n.d.).

In the CBO approach, CBOs in 22 counties received funding to deliver KIU! through their HIV prevention and testing programs. In the DTC approach, centrally-located staff recruited YMSM in 22 other counties using online advertising and shipped HIV self-tests and rectal and urethral chlamydia and gonorrhea sample collection kits (the latter two of which they were to ship to a lab for processing). In both approaches, after e-consenting to participation and providing documentation of an HIV-negative test result participants received access to KIU! Implementation assessments were based on RE-AIM and the Consolidated Framework for Implementation Research (CFIR) (Damschroder, Aron, et al., 2009a; Glasgow et al., 1999). Effectiveness outcomes included STI diagnoses, PrEP uptake, or self-reported condomless anal sex at 12-month follow-up (later abbreviated to 3-months, described later).

Due to the dual effectiveness and implementation foci, we needed to distinguish activities in the trial to properly attribute their effects on outcomes. Activities were either part of the *service* necessary for implementation (i.e., intervention content, delivery) or part of the *research* intended to assess intervention effectiveness (i.e., surveys, HIV/STI testing). All interested individuals were given access to the KIU! intervention but had to meet study eligibility criteria to complete the research components.

Research procedures were approved by the Northwestern University IRB; service components were considered non-human subjects research (Mustanski, Benbow, et al., 2025a; Mustanski, Saber, Jones, et al., 2023). All participants provided e-consent and/or verbal consent prior to enrollment in the research portions of the study.

### Assumptions and constraints

As no DHIs were commercially available at the time of the study, we made assumptions about how DTC delivery would occur in the “real world” while also considering the research constraints of our trial (e.g., timeline, county-level recruitment, sampling quotas, budget constraints) (Benbow et al., 2025). One major assumption was that those voluntarily seeking HIV prevention or sexual health services would not expect to be paid, based on other digital health applications focused on prevention and behavior change (e.g., mindfulness, fitness) were available in the App Store or Google Play (i.e., DTC). As such, at the beginning of the study incentives were not guaranteed, but participants were entered into raffles for $50 gift cards or sexual health products for completing research surveys. Other updates were made to increase pragmatism based on assumptions about real-world DHIs. Table 1 compares DTC delivery of KIU! during the effectiveness trial (KIU! 2.0) with features of the KIU! 3.0 implementation study as originally planned, and the final design after all changes were made.

### Data sources

Data came from weekly enrollment logs; budget spreadsheets and spending logs; secure participant text messages to our study; participant contact notes; and archived team meeting notes, e-mails, Data and Safety Monitoring Board (DSMB) reports, and memos to NIH program officers. Enrollment and recruitment data were analyzed descriptively. Below, we describe five phases of KIU! DTC delivery aligned with pivotal events and how implementation and study design evolved as we learned the limitations of what was feasible and achievable.

## Results

DTC participants were on average 26.0 years old, with 57.6% identifying as Black, Latino, or another racial/ethnic minority. All U.S. states except Alaska, Wyoming, and Maine were represented in the DTC arm with 33.1% of participants living in the South, 26.2% in the West, 24.4% in the Midwest, and 16.3% in the Northeast Census Region. Other DTC participant characteristics are available elsewhere (Mustanski, Benbow, et al., 2025b).

To characterize the phases of DTC delivery, each section includes high-level enrollment data and advertising spending; quotes from participants’ text messages or phone calls with DTC staff; and information from investigator meetings and conversations with NIH program officers that guided study change decisions.

The CBO and DTC teams each consisted of a co-investigator and two or three staff, respectively, who led arm-specific activities. The investigative team consisted of the PI, the two aforementioned co-investigators, and three implementation scientists, two health economists, a quantitative methodologist, and the software development team lead, all of whom operated across study arms. Staff analysts and software developers supported the investigative team and deployed the KIU! software and research surveys. During initial project meetings, details about CBO and DTC performance were not shared with the opposing teams to avoid contamination.

### Phase 1: Trial launch, October 2019-February 2020.

Enrollment began in October 2019. In contrast to the nationwide DTC pilot, the DTC team’s advertising strategy consisted of social media campaigns in ZIP codes within the 22 counties. Since financial incentives could not be guarantee, the advertisements focused on community benefits, opportunities to win sexual health products, and concerns about sexual health to create demand for research participation (Bassett et al., 2015; Macapagal et al., 2020). The advertising budget per DTC participant enrolled was smaller than in KIU! 2.0, where $7500 in advertising yielded 144 enrolled participants (~$52/participant); in KIU! 3.0, our initial budget was $27,000 for a goal of 2200 participants (~$12/participant).

The DTC team anticipated that enrollment targets would not be achieved via paid online advertising alone given the budget constraints and limited geographic catchment areas. To address this concern, the DTC team identified health departments, LGBTQ- or HIV-focused advocacy organizations, social groups, or universities in each county. The intention was to find local champions for KIU! whose “personal touch” may lead to increased enrollment in these counties (Damschroder, Banaszak-Holl, et al., 2009; Schon, 1963; Valente & Pumpuang, 2007). Community members were encouraged to distribute information about KIU! at their organizations with flyers and pamphlets. The team also partnered with an LGBTQ-focused marketing company who placed paid advertisements on dating apps.

**Figure 1:**
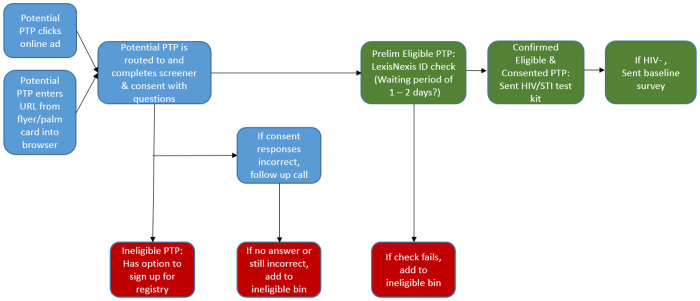
Participant Workflow during Study Phase 1

A challenge for the DTC team was to reduce obstacles to accessing KIU! (a pragmatic implementation goal) while deterring bots and imposter participants (a research goal) (Teitcher et al., 2015). Initially, a multi-step enrollment process was planned to deter imposters (Fig. 1).

After completing the eligibility survey, participant eligibility and mailing address was verified via a brief videochat or submission to a LexisNexis or WhitePages database, which would take 1–2 business days. Verified participants received an HIV/STI self-test kit in the mail. After participants submitted an HIV-negative test result to a secure online portal, they received the baseline survey, and then received the link to KIU! Participants were also asked to ship their STI samples to a lab. This yielded at minimum a 7–10 day lag between screener completion and receiving access to KIU!

Given the relative ease of enrollment in KIU! 2.0 and a large ad buy on dating apps that had accelerated prior study enrollment, we did not expect to have spent $10,744 to only enroll 15 participants – a significantly higher per-person spend ($716.27/participant) than in KIU! 2.0. We noticed significant attrition in the enrollment process. In February 2020, 55 people were preliminarily eligible, of which 38.2% (n = 21) were confirmed; 71.4% of these (n = 15) completed baseline and were enrolled (but only 27.3% of those preliminary eligible). Participants were required to answer comprehension questions about the consent form, and those who missed questions had to consent over the phone, which was a challenge to schedule: “I won’t be home until 11 tonight if that’s fine with you. Or you can call me before 9AM or after 7PM tomorrow, or anytime Saturday” (text message from participant, 11/07/19). Thus we brainstormed methods to streamline enrollment, such as shortening the screener and verifying respondents’ identity through social media and online directories rather than via LexisNexis.

### Phase 2: March 2020-June 2020.

**Figure 2:**
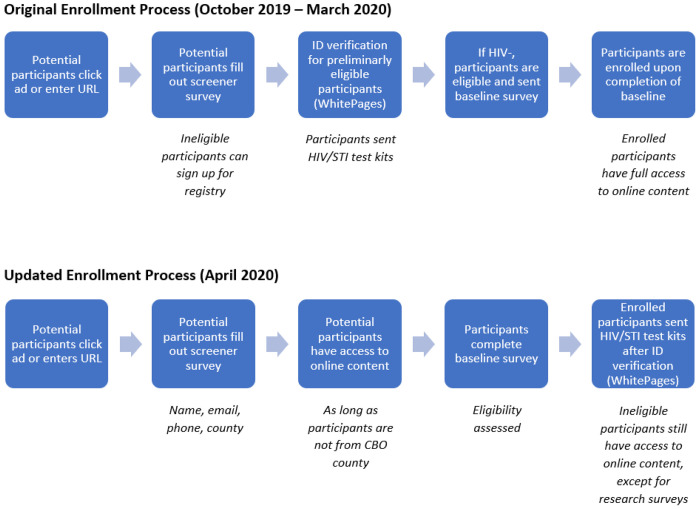
Comparison of Enrollment Processes Implemented during Study Phases 1 and 2

After the COVID-19 pandemic restrictions began, the DTC team paused HIV/STI test shipments because staff were not permitted at the university. Requiring proof of an HIV-negative test result would prevent enrollment, so DTC participants were allowed to enroll in the intervention based on a self-reported negative or unknown HIV status. The changes brainstormed in Phase 1 were implemented to streamline the enrollment process (Fig. 2). To incentivize participants, in consultation with the investigative team, the DTC team increased the value and frequency of the raffle prizes from a $50 raffle for every 50 participants who completed a survey (which meant that there were long delays between raffles) to monthly raffles for $200 regardless of how many participants were enrolled. These changes were intended to increase demand for participation and ensure we collected sufficient data to power our effectiveness analyses.

The investigative team had concerns about the impact of the pandemic on participants’ sexual behavior (and thus inclusion criteria) and related study outcomes (e.g., HIV transmission risk behavior). Participants shared that participating in KIU! 3.0 was no longer a priority as they dealt with health issues, employment and educational instability, and greater caretaking responsibilities. In response to a study reminder, one participant said, “Honestly with all the things going on [in] the world I just have so much else to focus [on]. I was laid off my job so looking for a job is my main priority” (04/03/2020). Another participant said: “It’s been really difficult the last couple of months dealing with school, mental health, family and other aspects of life and I’m trying to get back to the online material as soon as possible.” (06/12/2020).

In May 2020, staff could return to the office and a DTC team member resumed shipping of HIV/STI test kits every 2 weeks (vs. any time a participant enrolled) to balance team safety while continuing the study. We also contracted with a company who shipped HIV/STI test kits for us, which allowed greater flexibility for handling testing during COVID stay-at-home orders. Despite these adaptations, only 26 participants were enrolled at the end of this phase, and an additional $6,428 was spent on advertisements.

### Phase 3: July 2020-September 2020.

Several additional changes were implemented in this phase to increase demand for KIU! 3.0. We decided to guarantee incentives for completing episodes 1–3. To examine whether different incentives would result in varying engagement and retention, DTC counties were randomized to receive $10 or $25, modeled after prior research (Sharma et al., 2011). In August 2020 online recruitment on social media and dating apps’ self-service portals was supplemented by a marketing company experienced in online recruitment for health research studies. Snowball recruitment began as well, with enrolled participants allowed to refer up to five friends and receive $10 for each enrolled friend. Eligibility criteria were modified again in September 2020 to allow enrollment of gender-diverse participants assigned male at birth in recognition of the fact that sexual behavior, and not gender, drives HIV risk, and because in KIU! 3.0 and the team’s prior HIV prevention studies focused on cisgender men, nonbinary and gender-diverse participants comprised a significant proportion of ineligible individuals. Yet by the end of this phase, an additional $3,397 was spent on advertising, and DTC enrollment only increased to 64 participants. Individuals who had not finished the baseline survey were recontacted to increase enrollment, and feedback such as, “I didn’t complete [the survey] because I am out of your age range” (10/07/2020), was common, suggesting that additional modifications to eligibility criteria could benefit the study. In addition, only 52.8% of participants had returned their baseline STI samples to the lab.

### Phase 4: October 2020-February 2021.

By this time it was evident that the several changes made to the study only modestly improved enrollment. The investigative team decided to inform the co-investigators leading the CBO and DTC arms that both groups were similarly struggling with enrollment and that more significant changes to the study design were necessary to avoid trial failure (i.e., the inability to enroll enough participants to assess either effectiveness or implementation).

In October 2020, discussions began between the DTC team and implementation scientist co-investigators about expanding to nationwide recruitment. This change was deemed as most urgent because, out of all ineligible respondents for the DTC arm, nearly 25% failed screening due only to their county. This change also was perceived by the implementation scientist investigators to increase the DTC strategy pragmatism given that many commercially available DTC online health programs for other conditions are available more widely (e.g., via App Store, employer-sponsored insurance plans; Lau et al., 2020). However, this modification would fundamentally change the trial design (i.e., no longer cluster-randomized) and affect the ability to directly compare the effectiveness outcomes across arms due to the different scale of enrollment (i.e., county vs. national). Another change discussed was increasing incentives for completing the intervention content – not just the research surveys – to improve recruitment and retention. The investigative team believed this was justifiable as incentives were allowable in CDC funding opportunity announcements and found that it was common in public health practice to incentivize intervention completion (Zamantakis et al., 2025).

In addition to DTC-strategy-specific changes, the investigative team brainstormed changes that could be applied irrespective of CBO/DTC arm to facilitate enrollment and between-strategy comparisons despite changes to the RCT design. This included: 1) increasing the upper age limit from 29 to 34 following age groupings in CDC epidemiological reports and other studies that included 34 year olds among “YMSM” (Gerke et al., 2022; Hill-Rorie et al., 2024); 2) no longer requiring participants to have had condomless anal sex in the past 3 months; 3) making breaks between episodes 1–3 optional such that participants could complete most of KIU! in one sitting; 4) abbreviating the breaks between episodes 1–3 and 5 and 6; 5) abbreviating the follow-up period from 12 to 3 months; and 6) eliminating STI testing from the final follow-up. Participant contact notes revealed that breaks were an obstacle to sustained attention, motivation, and engagement in KIU!: “I’m also stuck in the waiting period for the next episode unless that ran out and I missed it because what even is time” (1/23/2021).

The DSMB and investigative team met in November 2020 to discuss the changes, which the DSMB supported and provided feedback regarding the impacts on aims, sample size, and safety. In December 2020, the proposed changes were submitted to the NIH program officers for approval. The program officers raised a concern about the request to drop STI testing at final timepoint as few HIV prevention studies at the time collected biomedical effectiveness outcomes; this resulted in retention of the STI testing endpoint. NIH and the university IRB approved all changes in February 2021. The study team spent the rest of this phase updating the KIU! materials, software, and advertising campaigns to reflect the changes. This phase ended with an additional $4,474 spent on advertising and DTC enrollment increased to 118 participants.

### Phase 5: March 2021 – March 2023 (end of trial).

When the changes proposed in Phase 4 went into effect, the DTC team launched national advertising campaigns on Facebook, Instagram, Snapchat, and Grindr and ran another campaign with the LGBTQ-focused marketing company. These were supplemented with offline strategies such as snowball recruitment, referrals from other studies, and participant registries. Those who were previously ineligible due to location were recontacted and informed about the new compensation structure: $50 for the first three episodes, $10 for the 6-week booster, and $15 for the 3-month booster. To facilitate adherence to STI testing at the 3-month endpoint, participants in both CBO and DTC strategies received an additional $10, for a total of up to $85 in incentives.

These changes had a sizable and rapid impact on enrollment. One month after the changes, DTC enrollment increased from 118 to 355 participants (a 301% increase). The average cost of advertising per enrolled participant also dramatically decreased from $212 to $79. Many participants noted the incentives as an important motivator: “I was actually only interested in the incentives” (6/17/2021). Another sent the text message, “ill do it soon i want the money” (3/29/2021). However, the incentives were associated with a significant increase in imposter participants, resulting in additional measures to verify participants’ identity and their survey data.

**Figure 3:**
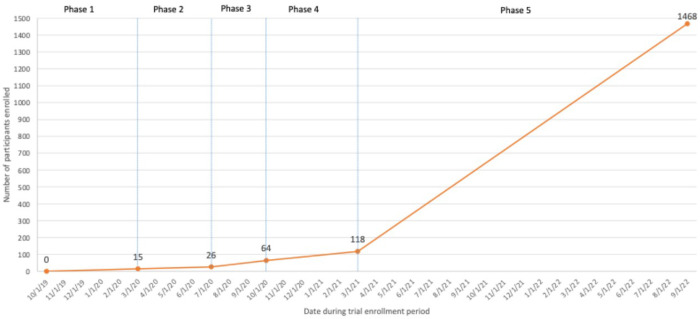
Participant enrollment through the five phases of the direct-to-consumer arm of the KIU! 3.0 hybrid effectiveness-implementation study, 2019-2022

After reallocating a substantial amount of the budget to advertising in the prior phases, and after deciding to guarantee incentives for both surveys and intervention completion, it became clear that the goal of enrolling 2,200 participants with the remaining budget and timeframe was infeasible. Thus, in the final phase NIH gave permission to reduce the DTC enrollment goal from 2,200 to a minimum of 1,100, and to extend the trial end date from April to December 2022. The previous sample size needed to power the cluster-randomized trial analyses was no longer necessary, and the new sample size would still provide meaningful data. The last participant was recruited in August 2022 yielding 1,468 participants enrolled in KIU! DTC. Across the trial, a total of $47,626 (~$32 per enrolled participant) was spent on DTC advertising, significantly more than the $27,000 budgeted, and over $100,000 was spent on incentives. Figure 3 depicts changes in DTC enrollment over the trial; Table 2 summarizes the barriers in each phase, solutions, and the impact on enrollment.

### Retention period.

Although enrollment in KIU! DTC improved, retention in the research study components remained a challenge even with incentives. First, retention at the 6-week and 3-month follow-up improved from 38% and 24%, respectively, to roughly 60% across both. In contrast, KIU! 2.0 had > 80% retention (Mustanski et al., 2018). The combination of intervention complexity, lower incentives, and the pandemic likely contributed to these lower rates. Also, participation in a study focused on preventing a health condition people did not have – or may not have felt the need for, given constrained opportunities for social and sexual contact in the early pandemic – was likely a lower priority based on communications between staff and participants. Yet final retention rates were still within ranges described in systematic reviews of digital health interventions, which demonstrate greater attrition in digital studies with larger samples, limited or no incentives, and limited or no human contact (Daniore et al., 2022; Torous et al., 2020).

Second, the return rate for STI testing at 3-month follow-up (primary effectiveness outcome) was 48% (n = 699/1468). This lower-than-expected return rate may be explained by the initial decision to send test kits only to participants who reconfirmed their mailing address prior to the 3-month follow-up. The intent was to save resources and avoid mailing kits to participants who had moved or gave an incorrect address. Eventually these test kits were mailed to all participants regardless of address confirmation. This increased return rates from 19% at the end of Phase 4 to 48% at the end of Phase 5.

After the last participant was enrolled, for the final 4 months of 2022, the DTC staff shifted into a retention-only phase, sending participants more frequent reminders to complete content and return STI testing kits via personalized calls, emails, and pre-recorded voice messages. Through this work, it became evident that participants thought that *all* retention reminders were automated. This led to an increase in receiving replies such as “STOP” or “UNSUBSCRIBE” via text. There was difficulty finding a middle ground between personalizing participant reminders for each participant’s situation and study behavior and automating them for efficiency in a large trial. Moreover, some participants seemed suspicious of study motivations when they perceived too many automated messages were sent, with one participant asking via a text: “How are you offering this [STI test] for free? Is my personal information being sold? Why do i need to complete a survey?” (12/01/2020)

## Discussion

Overall, the execution of this project reflected a constant tension between the pragmatic implementation aim (reaching as many people as possible in as real-life of a way as possible) versus the effectiveness aim and the deliverables required by the funders (collecting as much effectiveness outcome data as possible). These tensions often influenced the ability to make decisions that would best facilitate uptake and use of KIU! Although in principle, hybrid type 3 designs prioritize implementation over demonstrating effectiveness, at times it seemed both outcomes were equally important. An exclusive focus on studying implementation of an established effective intervention may have made space for different decisions more aligned with enhancing implementation success. Hybrid effectiveness-implementation study designs in theory accelerate the translation of research to public health practice but in actuality may constrain what can be learned about implementation, at least in the context of an intervention with time-limited external funding that expected collection of biomedical outcome data. Given the challenges in collecting primary data in large implementation trials, it has been suggested that these studies may instead rely on other sources (e.g., administrative, EHR data) as a proxy (Curran et al., 2012). Nevertheless, we hope that this narrative of our experience attempting to implement a DTC digital health intervention within a large hybrid type 3 study may pull back the curtain on what one might experience when doing similar studies of other digital interventions.

We offer several lessons learned for others planning to study implementation of a digital health intervention. First, we encourage investigators to consider what barriers to entry are critical (if any), including eligibility criteria, steps needed to access the intervention, and the time elapsed between screening eligible and entering the intervention, and we encourage investigators to do a real-time internal pilot of these steps if feasible. At the beginning of KIU! 3.0, study eligibility criteria were still relatively stringent, with multiple steps before participants could access the program. Although our cluster-randomized design was rigorous, ultimately it was not pragmatic for this intervention. In practice, one’s ability to access a self-guided, freely available digital intervention is rarely confined to the county they live in. These barriers, combined with our budget, constrained our ability to reach participants and led to significant attrition before people even accessed KIU! (Benbow et al., 2025). When research procedures (e.g., multistep screening, test kit verification, follow-up assessments) are the main barrier to entry, they can undermine implementation success, and may risk distorting what uptake might look like in real-world deployment.

Second, we encourage investigators to consider whether their participant incentives are sufficient for their implementation study. Given our past experiences with grateful participants and the literature on altruism as a motivator for health research participation (Williams et al., 2008), we overestimated participants’ willingness to participate and underestimated their interest in financial incentives. Eventually the DTC team learned that the CBOs implementing KIU! offered monetary incentives to participants as high as $100. Given that the CBO and DTC teams initially operated independently of each other to prevent contamination (Mustanski, Saber, Jones, et al., 2023), the DTC team was not aware of these incentives until the trial design was changed. The existence of incentivized HIV prevention programs may have led some participants to expect similar payments for KIU!, and potential participants told us that they lost interest once learning that raffle prizes were the only incentive.

In addition, guaranteed benefits likely play a role in reach and retention in implementation studies. In earlier KIU! evaluations, participants indicated that at-home STI testing was convenient as this preceded the rise of DTC health companies who now offer this service, and rectal STI testing was not yet widely available. Yet in this study, these same STI tests and the raffle prizes were insufficient to make study participation appealing. When asked about the reasons for not completing the STI tests, participants’ comments (e.g., “treatments are available”, “I only care about HIV”) suggested that STIs may be less of a concern within this population than HIV (Sarno et al., 2021) particularly as many STIs are treatable and preventable with antibiotics (Cannon & Celum, 2023). Moreover, since the KIU 2.0! trial, rectal STI testing has become more common in clinical settings, which may have made our provision of these tests less compelling. Providing incentives was valuable to study retention as well. Previous research has shown that user engagement with self-guided digital health apps is high initially, but few sustain engagement over time (Baumel et al., 2019). We addressed this issue by offering incentives for each survey and condensing the study timeline. Moreover, given that the study recruited during a time when many young people lost jobs, monetary incentives may have been more salient when making decisions about how they want to spend their time. Our economic analyses showed that even with these incentives, KIU! still represented a cost-saving intervention in the long term (Munroe et al., 2025; Mustanski, Benbow, et al., 2025b). Together these experiences reflect the concept of “conditional altruism” (McCann et al., 2010) – although altruism itself may have been part of participants’ calculus, offering the right combination of incentives and/or benefits is likely necessary to yield enrollment.

A third lesson learned is that dedicated staffing is a critical part of the implementation strategy for DTC self-guided digital interventions, particularly those that may require greater engagement to be effective. The adage “if you build it, they will come” did not hold true for our intervention, as simply advertising KIU! was not enough reach and retain people. Having a team who is skilled in reaching, engaging, and motivating participants from a distance can be the difference between successful and failed implementation of DTC digital interventions. Further, restrictions in audience targeting on self-service online marketing platforms (e.g., Meta), lower public trust in social media and online advertising (Park et al., 2020), and the increase in imposter participants in online research (Roehl & Harland, 2022) have made recruiting participants more challenging. Finally, although attrition in digital intervention studies (Daniore et al., 2022; Torous et al., 2020) is often high, if an implementation study requires effectiveness outcome data, retention is critical. Humanizing the intervention with personalized contacts and using techniques such as motivational interviewing to encourage study completion (Downs, 2019; Jake-Schoffman et al., 2021) enabled us to foster friendly relationships with study participants and troubleshoot barriers to study completion, but such frequent contacts were not always desired by participants.

Finally, unanticipated or underestimated outer setting factors (e.g., the pandemic, attitudes about the necessity of HIV/STI prevention) were major determinants of how the DTC strategy performed (Damschroder, Aron, et al., 2009b). However, researchers are not always equipped to deal with them, especially if they are newer to testing their interventions in implementation studies. One recommendation is to do a pre-mortem which may enable teams to better anticipate potential pitfalls and develop contingency plans in the event recruitment or retention does not go as planned (Wippold et al., 2025). Another is to test digital interventions in the context they were meant to be delivered from the very beginning (rather than beginning with traditional efficacy trials) (Beidas et al., 2023) which would enable teams to identify such roadblocks – and move interventions to public health practice – sooner.

## Conclusion

Hybrid type 3 effectiveness-implementation studies of DTC digital health interventions remain relatively uncommon. Our experience in this study was illuminating and has since informed our approach to other hybrid studies of digital and multimedia interventions. This study attempted to approximate real-world conditions, yet research constraints precluded it from achieving naturalistic implementation conditions, and investigators seeking to do similar work may encounter frequent tensions between research and implementation priorities. As digital health services continue to expand to real-world settings, investigators and funders alike may need to reconsider how hybrid designs are structured, from barriers to entry, the extent to which incentives and sustained engagement are important to answer one’s research questions, which outcomes are essential and how they can be easily captured for real-world use, to how researchers who may also be implementers may consider the multiple “hats” they may wear in a study (Curran et al., 2012). Careful consideration of these issues can help ensure that the pursuit of rigor does not inadvertently constrain the very implementation processes these studies aim to advance.

## Figures and Tables

**Table 1.** Eligibility and methods of KIU! effectiveness trial, vs. original design and final design of the hybrid study (DTC delivery only)

Component	KIU! 2.0 Effectiveness Trial	KIU! 3.0 Implementation-Effectiveness Study (original design)	KIU! 3.0 Implementation-Effectiveness Study (final design)
Eligibility			
Age	18–29	18–29	18–34
HIV status	Negative result from at-home HIV test kit	Negative result from at-home HIV test kit	Self-reported HIV negative or unknown status
Relationship status	Not in a monogamous/exclusive relationship lasting more than 6 months	No requirement	No requirement
Sexual risk behavior	Condomless anal sex with a male partner in prior 6 months	Condomless anal sex with a male partner in prior 6 months	No requirement
PrEP use	No requirement	No PrEP use OR not adherent in prior 6 months	No PrEP use OR not adherent in prior 6 months
Screening	Online screener	Online screener	Online screener
Location	Nationwide	United States with counties (n = 22) with large diverse YMSM populations	Nationwide
Primary recruitment strategies	Online ads	Online ads; participant registries; friend and community partner referrals	Online ads; participant registries; friend and community partner referrals
Recruitment budget	$7500 (total actual spend)	$27,000 budgeted	$47,626 (total actual spend)
Sample size	144 (actual enrollment from DTC pilot)	2200 (goal)	1468 (actual enrollment)
Delivery	Online; 24-hour wait between intervention modules	Online; 8-hour wait between intervention modules	Online; no wait between intervention modules
Compensation	Up to $180 for completing surveys, intervention content, and at-home STI testing	Entry into raffle for $50 for completing surveys	Up to $100 for completing surveys, intervention content, and at-home STI testing

**Table 2:** Challenges, solutions, and impact on sample size across the five phases of KIU! 3.0

Phase 1	Challenges	Solutions	Sample Size at Phase End
	Limitations of online recruitment Multistep eligibility screening and enrollment process	Supplement online recruitment with outreach to community stakeholders who can refer participants into study Shortened eligibility screener	15
Phase 2	COVID-19 pandemic limits staff working in the office and impacts HIV/STI testing Lengthy eligibility screener survey Low enrollment	At-home HIV and STI test kit preparation and shipments paused; third-party at-home testing option offered Participants allowed to self-report unknown or negative HIV status and enroll into study Registration form collecting basic contact and county info replaced the eligibility screener survey Raffle increased to $200	26
Phase 3	Low enrollment and intervention completion rates Restrictive eligibility criteria	Randomized incentive of $10 or $25 offered to participants who complete first three KIU! episodes Online ad campaign in collaboration with LGBTQ-specific marketing and advertising company Incentivized snowball recruitment Eligibility criteria updated: Gender diverse participants assigned male at birth eligible for study	64
Phase 4	Restrictive eligibility criteria, low enrollment and retention rates, forced breaks between intervention content, lengthy study timeline	Changes to eligibility criteria, study design, and incentives discussed and proposed to and approved by study DSMB, IRB, and NIH Updates made to KIU! app and databases to reflect these changes and to prepare for national recruitment campaigns	116
Phase 5	Restrictive eligibility criteria, low enrollment and retention rates, forced breaks between intervention content, lengthy study timeline	Largest changes made: 1. Expanding DTC catchment area to national recruitment. 2. De-emphasizing effectiveness and focusing on implementation aims. 3. Increasing upper age limits of eligibility from 29 to 34 years. 4. Removing the condomless anal sex eligibility criteria. 5. Making breaks between the first three sessions of the intervention optional. 6. Shortening period of follow-up from 12 months to 3 months. 7. Collecting follow-up data at one time point only (3 months). 8. Increasing the incentive paid to DTC participants who complete the first three sessions of the intervention from $10 - $25 to $50. 9. Paying DTC participants who complete their 6-week and 3-month follow-ups $10 and $15, respectively.	1468

## Data Availability

Limited data available upon request.
